# Rules have reasons: response to Greay et al. (2019)

**DOI:** 10.1186/s13071-020-04454-y

**Published:** 2020-11-11

**Authors:** D. James Harris

**Affiliations:** 1 Centro de Investigação em Biodiversidade e Recursos Genéticos (CIBIO), InBIO Laboratório Associado, Universidade do Porto, Campus Agrário de Vairão, 4485-661 Vairão, Portugal; 2 Departamento de Biologia, Faculdade de Ciências, Universidade do Porto, R. Campo Alegre s/n, 4169-007 Porto, Portugal

**Keywords:** *Babesia*, *Theileria*, *Hepatozoon*, Companion animals, Ticks

## Abstract

Recently Greay et al. (Parasit Vectors 11:197, 2018) described several new Apicomplexa parasites from domestic companion animals in Australia. Harris (Parasit Vectors 12;172, 2019) highlighted that these descriptions did not conform to the International Code of Zoological Nomenclature (ICZN) rules. Despite Harris (2019) clearly noting “molecular characters can be used to satisfy article 13.1.1 of the code”, in a reply Greay et al. (Parasit Vectors 12:178, 2019) incorrectly state “Harris considers the eight new species…invalid on the basis that only molecular characters were provided”. This was not the case. The ICZN has strict rules regarding species descriptions for good reasons. Here I reiterate why the forms described by Greay et al. (2018) are not valid.

## Letter to the Editor

Greay et al. [1] described eight novel Apicomplexan species from ticks taken from pets in Australia, based on 18S rDNA sequence data. Harris [2] reported that these did not conform to International Code of Zoological Nomenclature (ICZN) rules, in that new names must be accompanied by a description or definition stating *in words* the characters that differentiate them. Greay et al. [3] counter with several points. First, they note that codes and committees governing the nomenclature of viral and microorganisms have “largely adopted the use of sequence data to describe novel species”. This may be correct, but is irrelevant since protozoan classification falls under ICZN rules. Next they state that criticisms of DNA-based species descriptions have been refuted, and I essentially agree; however, this is not a discussion regarding systematic approaches but a determination of whether ICZN rules have been applied or not. Greay et al. [3] then propose that the text “see above”, which refers to the GenBank Accession numbers is sufficient to comply with the rules, and that the defined characters are the 18S rDNA sequences. They further note that journals have no standardized format for sequence descriptions, and that while some authors report specific nucleotide polymorphisms, this would be “unsuitable for large datasets (especially genomes)”. These arguments are, however, fallacious. Journal formatting, or dataset sizes, are irrelevant in cases of new species descriptions—the only issue is if ICZN rules were followed. 18S rDNA sequences are not characters, but suites of characters—the characters are individual nucleotide positions. In a similar fashion, a morphological diagram cannot alone be used to describe a new species, even if it encompasses all the key features (as an example, see [4]). Rule 13.1.1 directs that characters must be stated in words. Had Greay et al. [1] noted specific nucleotide characters in the sequences that differentiate the forms, as was proposed by Cook et al. [5], these could have been used to comply with the rules. They did not. Adding them as an additional file to their later response [3] does not change this. Other cases exist where taxonomists have tried to base new species on sequence divergence, and these have been considered *nomen nudum*, or unavailable, under the ICZN code (e.g. [6]) for the same reasons that Harris [2] identified. Furthermore, even if the whole sequence could be considered a character, this would mean that only completely identical sequences could be associated with this name—not very informative with genomic datasets, or with 18S rDNA where variants are well known within species (e.g. [7]), and even within individuals [8]. Therefore, the approach proposed by Greay et al. [3] does not comply with current rules, nor would be practical even if it did.

To conclude, scientists can debate species concepts and systematic approaches, but rules are necessary for taxonomic conformity. If scientists disagree with these rules, they can advocate future changes (e.g. [9]), but simply ignoring them only destabilizes taxonomy. Greay et al. [1] did not follow the ICZN rules, and therefore the names proposed are unavailable. Rather than incorrectly stating this is a problem with the use of molecular data, new valid names can be proposed to resolve this issue. Alternatively, the authors can submit a case to the ICZN to act as an arbitrator regarding whether rules were complied with or not.

## Data Availability

Not applicable.
